# Disseminated, fatal reactivation of bovine tuberculosis in a patient treated with adalimumab: a case report and review of the literature

**DOI:** 10.1007/s15010-024-02364-0

**Published:** 2024-08-14

**Authors:** Gioele Capoferri, Giovanni Ghielmetti, Bettina Glatz, Markus R. Mutke, Alexandar Tzankov, Roger Stephan, Peter M. Keller, Niklaus D. Labhardt

**Affiliations:** 1 Division of Infectious Diseases and Hospital Epidemiology, University Hospital Basel, Basel, Switzerland; 2 Section of Veterinary Bacteriology, Institute for Food Safety and Hygiene, Vetsuisse Faculty, University of Zurich, Zurich, Switzerland; 3 Department of Internal Medicine, University Hospital Basel, Basel, Switzerland; 4 Institute of Pathology and Medical Genetics, University Hospital Basel, Basel, Switzerland; 5 Division of Clinical Bacteriology and Mycology, University Hospital of Basel, Basel, Switzerland; 6 Division of Clinical Epidemiology, Department of Clinical Research, University Hospital Basel, Basel, Switzerland

**Keywords:** *Mycobacterium bovis*, Bovine tuberculosis reactivation, Tumour necrosis factor inhibitors, Adalimumab, Non-necrotizing granulomas

## Abstract

**Purpose:**

Tumor necrosis factor inhibitors (TNFi) are known to increase the risk of tuberculosis (TB) reactivation, though cases involving *Mycobacterium bovis* are rarely reported.

**Case presentation/results:**

We describe a case of disseminated TB with *M. bovis* in a 78-year-old woman with a negative Interferon-Gamma-Release Assay (IGRA), taking adalimumab due to rheumatoid polyarthritis, which resulted in a fatal outcome. The atypical clinical and histopathological features were initially interpreted as sarcoidosis. The case occurred in Switzerland, an officially bovine tuberculosis-free country. The whole genome sequence of the patient’s cultured *M. bovis* isolate was identified as belonging to the animal lineage La1.2, the main genotype in continental Europe, but showed significant genetic distance from previously sequenced Swiss cattle strains. In a literature review, four cases of bovine tuberculosis reactivation under TNFi treatment were identified, with pulmonal, oral and intestinal manifestations. Similar to our patient, two cases presented a negative IGRA before TNFi initiation, which later converted to positive upon symptomatic presentation of *M. bovis* infection.

**Conclusion:**

This case highlights the diagnostic challenges of TB in immunosuppressed patients, the limited sensitivity of IGRA, and the importance of considering TB reactivation even in regions declared free of bovine tuberculosis. Detailed patient histories, including potential exposure to unpasteurized dairy products, are essential for guiding preventive TB treatment before TNFi initiation.

## Introduction

The increased risk of tuberculosis (TB) reactivation following treatment with tumour necrosis factor inhibitors (TNFi) is well-documented [1, 2]. However, reactivation of *Mycobacterium tuberculosis* complex bacteria (MTBC) other than *M. tuberculosis* is rarely reported. Since infections with *Mycobacterium bovis* are often extrapulmonary and the typically caseating structure of granulomas in patients with immunosuppression may be missed, making the correct diagnosis can be a challenge.

## Case presentation

In April 2023, a 78-year-old Caucasian Swiss-born woman was admitted due to 10 kg weight loss, weakness, and intermittent night sweats that began in October 2022, progressively intensifying over subsequent months. Her medical history included a diagnosis of seronegative rheumatoid polyarthritis in November 2019, initially treated with methotrexate and subsequently leflunomide. In September 2021, leflunomide was discontinued due to persistent symptoms of polyarthritis, and adalimumab was started. Before immunosuppressive treatment in November 2019, the patient tested negative for interferon-gamma release assay (IGRA; QuantiFERON-TB-Gold plus), had a slightly reduced lymphocyte count of 700/µL and an unremarkable chest x-ray.

**Fig. 1:**
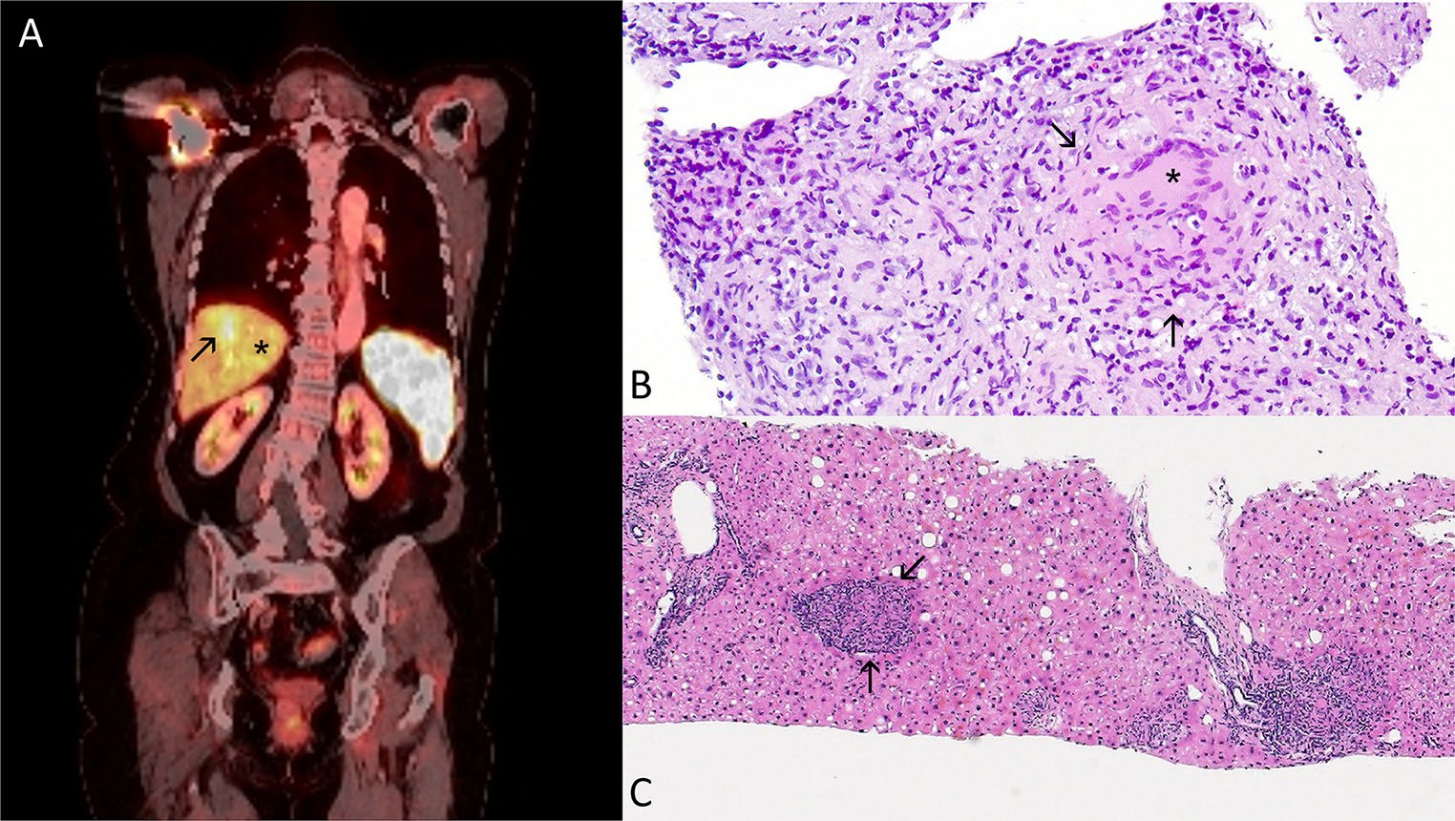
Radiological and histopathological findings. **a** PET scan showing disseminated hypodense, intensely hypermetabolic splenic lesions and two focal hypermetabolic areas in segments VIII (arrow) and III (*) of the liver. **b** Histopathologic findings in a splenic biopsy showing a granulocyte-admixed, vague granulomatous reaction (in-between arrows) with a giant cell (*) without necrosis (100x, hematoxylin and eosin stain). **c** Histopathologic findings in a liver biopsy showing granulomatous hepatitis with multiple rather small non-necrotizing granulomas (in-between arrows located in portal and lobular areas, consisting of lymphocytes, epithelioid and giant cells (40x, hematoxylin and eosin stain)

The work-up in April 2023 revealed heterogeneous echogenicity of the spleen. In Mai 2023, disseminated hypodense, intensely hypermetabolic splenic lesions, hypermetabolic hepatic lesions, small hypermetabolic bilateral pulmonary nodules, and intensely hypermetabolic lymph nodes at the hepatic hilus were observed in a positron emission tomography-computed tomography (PET-CT) scan (Fig. 1a). A biopsy of the hypermetabolic splenic lesions revealed non-necrotizing granulomatous splenitis (Fig. 1b). Polymerase chain reaction (PCR) targeting deoxyribonucleic acid (DNA) for mycobacteria species, *M. tuberculosis* and panfungal PCR from the formalin-fixed biopsy material yielded negative results.

In June 2023, the patient was again hospitalised due to deteriorating general condition, characterised by hypercalcaemia and acute renal failure. Upon suspicion of sarcoidosis, systemic corticosteroid therapy was initiated with subsequent clinical improvement. Further assessments for suspected sarcoidosis showed elevated angiotensin converting enzyme of 89 U/L, elevated soluble IL-2- receptor of 1996 pg/ml and elevated neopterin of 22.3 ng/ml. Due to low absolute lymphocyte numbers, the CD4:CD8 ratio from the broncho-alveolar lavage was inconclusive. Cultures for mycobacteria from the broncho-alveolar lavage were negative. Imaging showed no hilar adenopathy and biopsy of a hilar lymph node showed unspecific changes, i.e. lack of granulomas.

Serological investigations for the following infectious agents returned negative results: *Treponema pallidum*, human immunodeficiency virus, *Bartonella henselae*, *Coxiella burnetii*, *Francisella tularensis*, *Brucella* spp., and dimorphic fungi. A second IGRA (T-SPOT.TB) was clearly positive (ESAT-6 > 20, CFP-10 > 20). The lymphocyte count at that time was 770/µL. In May 2023, a biopsy of a hypermetabolic liver lesions seen in the PET-CT was taken to exclude a potential mycobacterial infection and revealed non-necrotizing granulomatous hepatitis (Fig. 1c). Due to the formalin fixation of the biopsy, mycobacterial culture was not performed, and PCR targeting DNA for mycobacteria species and for *M. tuberculosis* was negative. Blood cultures for mycobacteria were negative. Nevertheless, due to the differential diagnosis of mycobacterial infection, immunosuppression with steroids was gradually tapered and adalimumab was stopped in August 2023. Hypercalcaemia and renal failure recurred in September 2023. Treatment with steroids and adalimumab was re-initiated. One month later, in October 2023, the patient was readmitted for fever, dyspnoea, general weakness, disorientation and confusion. A computed tomography scan of the lung showed new bilateral infiltrates. Concurrently, one early-morning urine cultures for mycobacteria collected in September showed growth of MTBC. A repeated bronchoalveolar lavage showed acid-fast bacilli with PCR positive for MTBC, leading to the diagnosis of disseminated TB. Definitive culture results revealed *M. bovis*. Retrospectively, the patient reported that she had grown up and worked on a farm until about 30–40 years ago, where she drank unpasteurised cow milk, potentially exposing herself to *M. bovis*. Extensive medical history taking did not reveal any more recent potential exposures to *M. bovis* and the clinical course is consistent with an oral route of infection.

Standard treatment with rifampicin, isoniazid, ethambutol, and pyrazinamide was initiated. After identification of *M. bovis*, pyrazinamide was stopped. The patient, however, developed severe hepatitis, leading to a stop of the anti-tuberculosis therapy. After improvement of liver values, we restarted the therapy sequentially, first with rifampicin and then with isoniazid and ethambutol. Unfortunately, liver values increased again, leading us to replace isoniazid by moxifloxacin and switch rifampicin to rifabutin. However, the hepatopathy worsened, leading to complete treatment cessation after six weeks, considering the patient’s informed decision not to continue the therapy at this point. The patient passed away in November 2023.

**Fig. 2:**
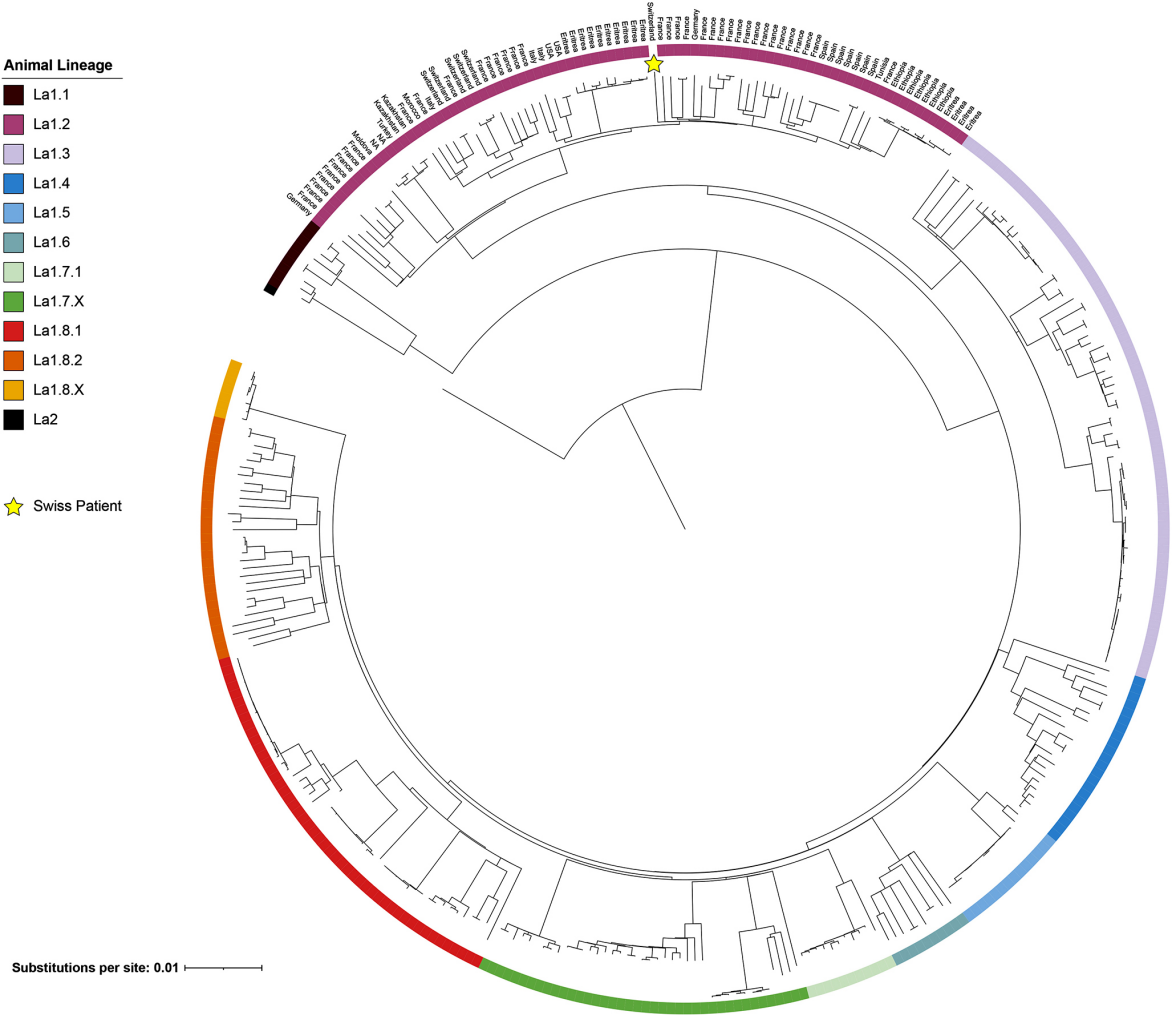
Maximum-likelihood phylogenetic tree generated with the GTR-CAT substitution model including the whole genome sequence of the *M. bovis* isolate cultured from a urine sample of a Swiss patient, is presented. A total of 350 sequences representing the currently known 8 lineages of *M. bovis* are color-coded, and the country of origin of the isolates identified as La1.2 is reported. The tree was rooted using *Mycobacterium caprae*, and the bar scale represents the number of substitutions per site

Using validated bioinformatic pipelines [3], the whole genome sequence (Illumina based sequenced) of the cultured isolate was determined to belong to the animal lineage La1.2 (raw sequencing data are available at European Nucleotide Archive under the accession number PRJEB75913) [4]. To contextualize this finding with previously isolated *M. bovis* strains from Switzerland and other countries, a total of 350 genomes covering the currently known 8 lineages of *M. bovis* were obtained from public repositories. Figure 2 shows the overall single-nucleotide polymorphism(SNP)-based phylogenetic tree including the patient’s *M. bovis* sequence and five previously sequenced genomes from Swiss cattle and one human patient obtained between 2000 and 2014. Notably, all Swiss *M. bovis* sequences belong to the La1.2 lineage, one of the main genotypes circulating in continental Europe. However, the sequence from the present study was genetically distant (approximately 330–450 SNPs) from previously sequenced Swiss *M. bovis* strains from cattle [5].

## Discussion

We present a fatal case of disseminated *M. bovis* infection, which was most likely due to reactivation after exposure several decades ago in a patient under medical immunosuppression. The patient tested negative on IGRA prior to the start of immunosuppression, but subsequently converted to a positive IGRA test. This case highlights three important clinical implications. Firstly, it is important to note that a negative IGRA test before starting medical immunosuppression may not definitively rule out MTBC infection due to its limited sensitivity of around 80–90% [6]. Secondly, TB can manifest atypically under TNFi, with non-caseating granulomas in biopsies and transient clinical response to corticosteroids. Thirdly, even in countries that have been considered free of *M. bovis* for decades, bovine tuberculosis (bTB) remains a differential diagnosis among the elderly population.

Since 1959, the Swiss cattle population has been officially recognised bTB-free according to article 8.11.4. of the terrestrial code of World Organisation for Animal Health [7], with only sporadic cases reported thereafter [5]. The rarely occurring human infections with *M. bovis* in Europe [8] mainly affect the elderly populations with a history of consumption of unpasteurized dairy products in childhood. Due to the primarily extrapulmonary manifestation [9] and frequent occurrence among older people with co-morbidities [10], diagnosis may be delayed, as was the case in our patient. Of particular note is the potential atypical histological manifestation under TNFi, characterized by disorganized granulomas without necrosis, contributing to delayed diagnosis or leading to a misdiagnosis of sarcoidosis. In vitro studies described atypical histological presentation with disorganized granulomas under TNFi [11–13]. Several clinical reports, however, describe the formation of caseating granulomas in patients treated with TNFi [14–16]. In low-income settings, the proportion of *M. bovis* infection among human tuberculosis cases is often higher compared to high-income countries, accounting for up to 30% of active human TB cases [17], although the exact prevalence remains unclear [18].

A literature search through PubMed revealed four case reports of *M. bovis* reactivation under TNFi treatment, including one oral, one pulmonary, and two intestinal manifestations (Table 1) [15, 16, 19, 20]. Similarly to our patient, two report a negative IGRA before initiation of infliximab, which converted positive when the patient became symptomatic for the *M. bovis* infection. The sensitivity of the IGRA is estimated at around 80% and slightly higher with T-SPOT^®^.TB as compared to QuantiFERON^®^-TB Gold In-Tube or tuberculin skin test [6].

**Table 1:** Cases of infection with *Mycobacterium bovis* associated with TNFi

Article, year of publication	Age	Sex	Country	Comorbidity	Test for LTBI before TNFi	Immunosuppression	Manifestation of tuberculosis	Repeated test for tuberculosis	Histology	Treatment and outcome	Probable exposure to *M. bovis*
Larsen et al. [19], 2008	79	F	Denmark	RA	TST negative, 2nd generation QFT indeterminate, Chest radiograph negative	Infliximab	Pulmonal noduli and pleural effusion, lather ascites	TST negative, 3rd generation QFT positive	Not done	RIF/INH/EMB for 9 months, cured	Worked at a diary during her childhood with exposure to unpasteurised milk
Nager et al. [15], 2009	69	F	Switzerland	Crohn disease	QFT negative (under Azathioprine), Chest radiograph negative	Infliximab	Ascites and extensive peritoneal inflammation	QFT negative, TST negative, T-SPOT.TB positive	Caseating granulomas (peritoneal biopsy)	RIF/INH/EMB for 3 months, RIF/MFX for 5 months, cured	Consumption of unpasteurized milk in a rural area of Switzerland during the 1940s
Ernst et al. [16], 2010	70	F	Germany	RA	Not reported	Etancercept, Adalimumab	Oral exulcerative mucositis and cheilitis	QFT slightly positive	Caseating granulomas (mucosal biopsy)	RIF/INH/ EMB/MFX for 2 months, RIF/EMB for 7 months, cured	Often at a family dairy during the 1940s and 1950s. She had been treated for „lung disease“ as a schoolgirl
Rodriguez-Orozco et al. [20], 2016	32	M	Mexico	RA	TST negative, Chest radiograph negative	Infliximab	Extensive ascites and pleural effusion	QFT positive	Not done	RIF/INH/EMB for 2 months, RIF/INH for 7 months, cured	Not clear
Our case, 2024	78	F	Switzerland	RA	QFT negative, Chest radiograph negative	Adalimumab	Lesions of spleen and liver, lather lung involvement (miliary)	T-SPOT.TB positive	Non caseating granulomas	RIF/INH/EMB for 1 months, death	Had grown up and work at a diary during 1940s and 1950s, when she drunk unpasteurised milk

LTBI, latent tuberculosis infection; TNFi, tumor necrosis factor inhibitors; RA, rheumatoid arthritis; TST, tuberculin skin test; QFT, QuantiFERON^®^ -TB test; T-SPOT.TB, T-SPOT^®^.TB-Test; RIF, rifampicin; INH, isoniazid; EMB, ethambutol; MFX, moxifloxacin

Conditions linked to lower sensitivity of IGRA are advanced age and low peripheral lymphocyte counts [21]. In our patient, repeating the IGRA test when she became symptomatic, even though she did not report any recent exposure to TB, was helpful, and the positive result was important to prioritize further work-up for mycobacterial infection.

Two of the four case reports mentioned above include histologic findings with both caseating granulomas. In our patient, in both the spleen and liver biopsies taken at different times, non-caseating disorganized granulomas were observed, no acid-fast bacilli were observed and the PCR results were negative at both occasions. TNFi, particularly monoclonal antibody preparations like infliximab and adalimumab, are considered to carry a moderate risk of reactivating latent tuberculosis (2). Thus, even after a negative IGRA, clinicians should not exclude TB in patients with possible mycobacterial infection, despite atypical histological presentations.

## Conclusion

In conclusion, this case emphasizes the diagnostic challenge of differentiating mycobacterial infections from other granulomatous diseases, such as sarcoidosis. It highlights the need to consider mycobacterial reactivation in immunosuppressed patients, particularly those treated with TNFi, even if the initial IGRA was negative and the presentation seems atypical. Furthermore, it is important to be aware of bTB epidemiology and to take a thorough history of unpasteurized dairy product consumption. This information may guide the preventive treatment of latent TB before initiating treatment with TNFi, even if the IGRA result is negative.

## Data Availability

No datasets were generated or analysed during the current study.
